# Feasibility analysis of pharmacy practice based on the evidence-based pharmacy model in the treatment of patients with severe pneumonia

**DOI:** 10.12669/pjms.41.11.12232

**Published:** 2025-11

**Authors:** Mingyue Liu, Jiayun Liu, Kang Meng, Yujia Ji, Lili Lu

**Affiliations:** 1 Mingyue Liu Department of Pharmacy, Baoding No.1 Central Hospital, Baoding 071000, Hebei, China; 2 Jiayun Liu Department of Anesthesiology, Baoding No.1 Central Hospital, Baoding 071000, Hebei, China; 3 Kang Meng Department of Pharmacy, Baoding Maternal and Child Health Hospital, Baoding 071000, Hebei, China; 4 Yujia Ji Department of Pharmacy, Baoding No.1 Central Hospital, Baoding 071000, Hebei, China; 5 Lili Lu Department of Pharmacy, Baoding No.1 Central Hospital, Baoding 071000, Hebei, China

**Keywords:** Evidence-based pharmacy, Severe pneumonia, Pharmacy practice, Anti-infective treatment

## Abstract

**Objective:**

To investigate the application of pharmacy practice based on the evidence-based pharmacy (EBP) model in the anti-infective treatment of patients with severe pneumonia.

**Methodology:**

This was a retrospective study. A total of 200 patients diagnosed with severe pneumonia caused by multidrug-resistant Gram-negative bacilli(MDR-GNB) and admitted to Baoding No.1 Central Hospital from April 2023 to March 2024 were randomly assigned to a control group(received conventional anti-infective treatment) and an experimental group(in addition to the conventional treatment, received pharmacy practice interventions based on the EBP model implemented by clinical pharmacists), with one hundred cases in each group.The two groups were compared in terms of antimicrobial drug use intensity, pre-antibiotic microbiological testing rates, clinical efficacy, adverse reaction rates, and irrational drug use.

**Results:**

The antimicrobial drug use intensity in the experimental group was significantly lower than that in the control group (*P<* 0.05), while the pre-antibiotic microbiological testing rate was significantly higher (*P<* 0.05). The antimicrobial drug costs and anti-infective treatment duration in the experimental group were lower than those in the control group (*P<* 0.05, respectively). The bacterial eradication and overall response in the experimental group were significantly higher than those in the control group (*P<* 0.05, respectively). Furthermore, adverse reaction and irrational drug use rates were significantly lower in the experimental group compared to the control group (*P<* 0.05, respectively).

**Conclusion:**

EBP model-based pharmacy practice may assist physicians in optimizing anti-infective treatment regimens for patients with severe pneumonia caused by MDR-GNB, improving treatment outcomes and medication safety while effectively enhancing patient prognosis.

## INTRODUCTION

Severe pneumonia is a serious respiratory infectious disease, often accompanied by severe hypoxemia or acute respiratory failure. In severe cases, it may present with shock and other manifestations of circulatory failure, as well as multi-organ dysfunction, leading to a high incidence and mortality rate.^1^ With the continuous increase in bacterial resistance, the anti-infection treatment of multidrug-resistant bacteria-associated severe pneumonia faces significant challenges. This results in longer hospital stays and significantly higher costs for treating resistant bacteria. Rational selection of antimicrobial agents and individualized dosing have become crucial for treating severe pneumonia.^2^,^3^ Research shows that Gram-negative bacteria (GNB) are the primary bacterial cause of severe community-acquired pneumonia (CAP) and hospital-acquired pneumonia (HAP), accounting for 50%–80% of infections.^4^

The detection rate of MDR-GNB has been increasing annually.^5^ The traditional empirical anti-infection treatment model is no longer sufficient to meet clinical needs, highlighting an urgent demand for more scientific and precise treatment strategies. Pharmacy practice based on the evidence-based pharmacy (EBP) model, conducted by clinical pharmacists specializing in anti-infection treatment, provides scientific evidence for individualized drug therapy based on the latest evidence in clinical medicine, combined with the pharmacist’s professional knowledge and the patient’s specific condition. This study aimed to explore the application of EBP model-based pharmacy practice in the treatment of MDR-GNB-induced severe pneumonia, providing scientific evidence for the precise treatment of the condition and improving patient prognosis.

## METHODOLOGY

This was a retrospective study. A total of 200 patients diagnosed with MDR-GNB-induced severe pneumonia and admitted to Baoding No.1 Central Hospital from April 2023 to March 2024 were selected for this study. Grouping and Intervention Patients were randomly assigned to a control group and an experimental group using a random number table, with one hundred cases in each group. The control group received conventional anti-infection treatment administered by clinical physicians. The experimental group, in addition to conventional anti-infection treatment, received EBP model-based pharmacy practice, implemented by clinical pharmacists.

### Ethical Approval:

The study was approved by the Institutional Ethics Committee of Baoding No.1 Central Hospital (No.:[2023]083; Date: March 04, 2023), and written informed consent in the was provided by all the individuals participating in the study.

### Inclusion criteria:

- Patients aged ≥18 years who meet the diagnostic criteria for severe pneumonia according to the *Chinese Adult Community-Acquired Pneumonia Diagnosis and Treatment Guidelines (2016 Edition)*.
- Patients with positive sputum/pulmonary lavage culture or pathogen microbiological second-generation sequencing results detecting multidrug-resistant Gram-negative bacilli (MDR-GNB), such as Klebsiella pneumoniae, *Acinetobacter baumannii*, Pseudomonas aeruginosa, and Escherichia coli.^6^
- Expected survival ≥7 days.
- Complete clinical data.
- Voluntary participation in the study with informed consent, able to communicate effectively with the researchers and comply with the study requirements.

### Exclusion criteria:

- Presence of other site infections.
- Impairment in consciousness or communication.
- Severe liver or kidney dysfunction.
- Hospital stay <48 h or withdrawal from the study for other reasons.

The clinical pharmacists used the latest evidence in clinical medicine, pharmacokinetics/pharmacodynamics (PK/PD) principles, and the patient’s pathophysiological characteristics and laboratory results as the basis for identifying urgent medication-related issues. They considered the distribution of pathogens and resistance patterns of patients with severe pneumonia in the hospital, in collaboration with the physicians, to discuss the anti-infection treatment plan. Based on the patient’s physical condition, infection severity, and pathogen characteristics, the pharmacists reviewed antimicrobial prescriptions/orders, conducted individualized pharmaceutical care, and evaluated the effectiveness of the anti-infection treatment.

The pharmacists performed dynamic analysis and evaluation of the rationality and safety of the anti-infection regimen, provided pharmaceutical advice for precise treatment, and assisted physicians in optimizing the medication regimen. This included selecting appropriate anti-infective drugs, determining the proper dosage, and setting the correct administration duration, aiming to achieve the best clinical treatment or infection prevention outcomes. While ensuring the therapeutic effects of anti-infection treatment, the pharmacists also conducted pharmaceutical care and medication education, focusing on potential adverse drug reactions or events. For clinical drug therapies with a narrow therapeutic index or those requiring precise drug dosage based on laboratory data, drug concentration monitoring was performed to prevent and reduce adverse reactions and improve the prognosis of patients with severe pneumonia. The follow-up period for both groups was six months.

### Outcome Measures:

- The following baseline data were collected: general patient information (*e.g*., sex, age, body mass index [BMI]), underlying diseases (*e.g*., hypertension, diabetes, coronary heart disease, chronic heart failure, chronic pulmonary diseases, malignant tumors), and antimicrobial drug exposure (defined as the use of the same antimicrobial drug for more than five days within the month prior to the onset of the illness).
- The two groups were compared in terms of pre-antibiotic microbiological testing rates, antimicrobial drug use intensity (*i.e*., defined daily dose), antimicrobial drug costs and treatment duration, clinical efficacy, bacterial eradication rate (BER), irrational drug use, and adverse reactions.
- *Criteria for Efficacy Evaluation:* Body temperature and respiratory symptoms were recorded as parameters for clinical efficacy evaluation. The clinical efficacy was classified into three categories: i) Significant Response (SR): Symptoms resolved, and signs improved by ≥75%, with normal laboratory results and no relapse. ii) Partial Response (PR): Symptoms were alleviated, and signs improved by 50%–74%, with laboratory results largely normalized *. iii) No Response (NR):* No symptom improvement or disease exacerbation. ORR = (SR cases + PR cases) / Total number of cases ×100
- *BER Calculation:* After treatment, qualified sputum/pulmonary lavage specimens were sent for microbiological culture to compare the difference in pathogenic bacterial eradication rates between the two groups *. i) Complete Eradication (CE):* No original pathogen was detected in the sputum/pulmonary lavage culture after treatment. *ii) Partial Eradication (PE):* Pathogens were reduced but not fully eliminated, with some remaining detectable in the sputum/pulmonary lavage culture after treatment. iii) No Eradication (NE): The original pathogen was still present in the sputum/pulmonary lavage culture and had not turned negative after treatment. iv) Replacement: The original pathogen was cleared in the sputum culture, but new pathogens were detected after treatment. *v*) Reinfection: New pathogens were detected in the sputum culture after treatment, indicating a reinfection. BER = (CE cases + PE cases) / Total number of cases ×100%
- The anti-infection medication regimens for both groups were summarized to statistically analyze and compare irrational drug use in both groups, such as inappropriate drug combinations, incorrect dosage and administration, unsuitable solvents, use without indications, prolonged treatment duration, and non-compliant use of high-level antimicrobial drugs.
- *Adverse Reactions:* The number of cases of adverse reactions, their specific manifestations, pharmaceutical interventions, and outcomes were documented, and the adverse reaction rates were compared between the two groups.

### Statistical Analysis:

Statistical analysis was performed using the software SPSS 26.0. Measurement data were expressed as mean ± standard deviation (*χ̅*±*S*) and analyzed using the t-test. Categorical data were expressed as percentages (%) and analyzed using the chi-square (*χ*²) test. A p-value <0.05 was considered statistically significant.

## RESULTS

Six patients were excluded due to poor compliance or lack of cooperation with treatment, resulting in 194 patients with MDR-GNB-induced severe pneumonia completing the study. There were no significant statistical differences between the two groups in sex, age, BMI, underlying diseases, and history of antimicrobial drug exposure, suggesting comparability (Table-I). The experimental group had a shorter duration of anti-infective treatment, lower antimicrobial drug use intensity, and reduced antimicrobial drug costs compared to the control group. All these differences were statistically significant (*P <* 0.01, respectively) (Table-II). The ORR in the experimental group was significantly higher than that in the control group (*P <* 0.05) (Table-III). The experimental group exhibited a significantly higher BER in post-treatment sputum/alveolar lavage fluid specimens compared to the control group (*P <* 0.05) (Table-IV).

**Table-I:** General patient information (*n* = 194).

Group	Case (n)	Sex				Age (years, χ̅±S)	BMI (kg/m², χ̅±S)	Underlying diseases		History of antimicrobial drug exposure	
		Male		Female							
		Case (n)	%	Case (n)	%			Case (n)	%	Case (n)	%
Control group	97	59	60.82	38	39.18	76.32±8.15	23.64±4.29	69	71.13	56	57.73
Experimental group	97	52	53.61	45	46.39	74.69±7.97	24.31±4.76	73	75.26	61	62.89
t/χ² value	1.46					1.23	1.02	0.67		0.89	
P-value	0.227					0.220	0.309	0.413		0.346	

**Table-II:** Comparison of antimicrobial drug use between the two groups (*n* = 194).

Group	Case (n)	Pre-antibiotic microbiological testing rate		Antimicrobial drug use intensity	Anti-infective treatment duration (days)	Antimicrobial drug costs (CNY yuan)
		Case (n)	%			
Control group	97	60	61.86	116.42±6.16	21.04±4.36	5919.68±84.37
Experimental group	97	95	97.94	104.57±5.29	19.38±4.01	5313.54±69.92
t/χ² value		45.68		12.34	3.21	7.89
P-value		<0.01		<0.01	<0.01	<0.01

**Table-III:** Comparison of clinical efficacy between the two groups (*n* = 194).

Group	Case (n)	SR	PR	NR
Control group	97	46(47.42)	35(36.08)	16(16.50)
Experimental group	97	57(58.76)	33(33.99)	7(7.22)
t/χ² value		4.12		
P-value		0.042		

**Table-IV:** Post-treatment BERs in the two groups (*n* = 194).

Group	Case (n)	CE	PE	NE	Replacement	Reinfection
Control group	97	46(47.42%)	27(27.84%)	16(16.49%)	5(5.15%)	3(3.09%)
Experimental group	97	55(56.70%)	31(31.96%)	7(7.22%)	3(3.09%)	1(1.03%)
*t*/*χ*² value		9.49				
*P-*value		0.002				

During anti-infective treatment, there were 17 instances of irrational drug use in the control group, including inappropriate dosage (*n* = 6), inappropriate combination therapy (*n* = 5), use despite contraindicated drugs (*n* = 2), and inappropriate drug selection (*n* = 4). In contrast, the experimental group had only seven instances of irrational drug use, which was significantly lower than the control group (*P <* 0.05). Regarding adverse reactions, 16 patients in the control group experienced adverse effects, while seven patients in the experimental group had adverse reactions, suggesting a statistically significant difference between the two groups (*P <* 0.05) (Table-V).

**Table-V:** Analysis of irrational drug use and medication safety in the two groups (*n* = 194).

Group	Case (n)	Irrational drug use				Adverse reactions				
		Inappropriate dosage and administration	Inappropriate combination therapy	Use of contraindicated drugs	Inappropriate drug selection	Reinfection	Drug-induced liver injury	Drug-induced kidney injury	Nervous system-related adverse reaction	Other adverse reactions
Control group	97	6(6.19%)	5(5.15%)	2(2.06%)	4(4.12%)	3(3.09%)	4(4.12%)	2(2.06%)	5(5.15%)	2(2.06%)
Experimental group	97	3(3.09%)	2(2.06%)	0	2(2.06%)	2(2.06%)	3(3.09%)	1(1.03%)	1(1.03%)	0
t/χ² value		6.25				5.02				
P-value		0.012				0.025				

## DISCUSSION

6In this study, EBP model-based pharmacy practice, conducted by clinical pharmacists, provided precise pharmaceutical advice to patients, assisting physicians in optimizing anti-infective treatment regimens by selecting appropriate antimicrobial agents, dosages, and treatment durations. This approach improved therapeutic efficacy and reduced adverse reactions.^7^,^8^ The study found that the experimental group outperformed the control group in antimicrobial drug use intensity, pre-antibiotic microbiological testing rates, antimicrobial drug costs and treatment duration, clinical efficacy, and bacterial eradication rates. Notably, the experimental group reported no contraindicated drug use, off-label use of nebulized polymyxin B monotherapy for MDR-GNB-induced severe pneumonia, or other irrational drug use.

Furthermore, during pharmacy practice, clinical pharmacists provided individualized medication education to patients, which improved their understanding and adherence to the treatment regimen, thereby reducing the incidence of adverse drug reactions caused by inappropriate drug use.^9^-^10^ Currently, the resistance of major pathogens causing severe pneumonia is increasing annually. MDR-GNB primarily includes pathogens that produce extended-spectrum beta-lactamases, AmpC beta-lactamases, and carbapenemases, such as carbapenemase-producing Pseudomonas aeruginosa, and carbapenemase-producing *Acinetobacter baumannii*.^11^ New antibiotics targeting these infections include ceftazidime-avibactam, cefiderocol, ceftolozane-tazobactam, meropenem-vaborbactam, imipenem-cilastatin-relebactam, eravacycline, and plazomicin.^12^,^13^ In China, the treatment of patients with MDR-GNB-induced severe pneumonia mainly involves the use of monotherapy or combination therapy with carbapenem, polymyxin, tigecycline, and ceftazidime-avibactam.^14^

However, due to the limitations of pharmacological properties, these drugs are often insufficient to meet the treatment needs of most patients. For example, polymyxin has poor pulmonary penetration, and the concentration of polymyxin in lung tissues after intravenous administration is low, making it difficult to reach effective therapeutic concentrations. It is also prone to heterogeneous resistance, and increasing the systemic dose can lead to an increased risk of nephrotoxicity and neurotoxicity. Inhaled polymyxin can significantly increase the drug concentration in lung tissue and reduce the incidence of nephrotoxic adverse events.^15^-^17^

Currently, there is no inhalation formulation available in China, and the nebulized inhalation of the injectable form constitutes off-label use. Tigecycline, at the standard recommended dose, has low lung tissue concentrations and poor penetration across the blood-brain barrier. In recent years, resistance has increased, with the minimum inhibitory concentration showing an upward trend. Increasing the dose could increase the risk of drug-induced liver injury, coagulopathy, and other adverse reactions.^18^ Ceftazidime-avibactam has low concentrations in lung tissues and is ineffective against metallo-β-lactamase-producing strains.

The resistance has been rising year by year since its market introduction.^19^ Pharmaceutical services based on the EBP model emphasize the integration of the best research evidence, clinical experience, and patient needs. Through the full involvement of clinical pharmacists, and by referring to the latest evidence in evidence-based medicine, common antimicrobial PK/PD principles, the patient’s specific pathophysiological characteristics, and laboratory findings, individualized medication issues that need to be addressed are clearly identified. Based on the current antimicrobial resistance status of multidrug-resistant pathogens causing severe pneumonia in our institution, clinical pharmacists work with physicians to discuss anti-infective treatment plans. Pre-treatment reviews are conducted according to the patient’s physical characteristics, infection severity, and pathogen detection. Personalized pharmaceutical care is provided during the treatment process, including dynamic evaluation of anti-infective efficacy. The rationality and safety of the anti-infective regimen are analyzed and assessed in real time, providing pharmaceutical advice for the precision treatment of the patient. This collaborative approach is particularly important for optimizing clinical treatment strategies.^20^

### Limitations:

However, the current research on the application of the EBP model in the treatment of MDR-GNB-induced severe pneumonia is still limited, and its specific effects and potential for broader application need further verification. It is recommended that future studies expand the sample size and conduct additional feasibility research based on our findings.

## CONCLUSIONS

This study demonstrated the significant value of EBP model-based pharmacy practice in the anti-infective treatment of patients with severe pneumonia, offering new insights into the treatment of MDR-GNB-induced pneumonia.

### Authors’ Contributions:

**ML:** Carried out the studies, drafted the manuscript and are responsible and accountable for the accuracy or integrity of the work.

**JL** and **KM:** Performed the statistical analysis and participated in its design.

**YJ** and **LL:** Collected the data, performed the analysis, critical review, were involved in the writing of the manuscript.

All authors have read and approved the final manuscript.
